# Combined EEG-fMRI and tractography to visualise propagation of epileptic activity

**DOI:** 10.1136/jnnp.2007.125401

**Published:** 2007-12-20

**Authors:** K Hamandi, H W R Powell, H Laufs, M R Symms, G J Barker, G J M Parker, L Lemieux, J S Duncan

**Affiliations:** 1 Department of Clinical and Experimental Epilepsy, UCL Institute of Neurology, Queen Square, London, UK; 2 Institute of Psychiatry, Kings College London, London, UK; 3 Imaging Science & Biomedical Engineering, The University of Manchester, Manchester, UK

## Abstract

In a patient with refractory temporal lobe epilepsy, EEG-fMRI showed activation in association with left anterior temporal interictal discharges, in the left temporal, parietal and occipital lobes. Dynamic causal modelling suggested propagation of neural activity from the temporal focus to the area of occipital activation. Tractography showed connections from the site of temporal lobe activation to the site of occipital activation. This demonstrates the principle of combining EEG-fMRI and tractography to delineate the pathways of propagation of epileptic activity.

An appreciation of the propagation of epileptic activity is key to understanding the basis of networks that underpin epilepsy. EEG-fMRI can localise the haemodynamic correlates of interictal epileptiform discharges (IEDs).^1^ Diffusion tensor imaging (DTI) evaluates the passive diffusion of water molecules in tissue, from which connectivity between voxels can be inferred.^2^ Probabilistic tractography algorithms generate maps of probability of connection from chosen start points,^3^ which can be displayed to visualise white matter tracts.^3^

Effective connectivity is a term describing the functional interaction of regions of the brain, when responding to a stimulus or performing a task. Dynamic causal modelling (DCM) is a statistical method for determining the functional interaction between specified brain areas that may be applied to fMRI data, and thus infer whether changes at one region are driving changes at another.^4^ The DCM methodology relies on a model of the relationship between fMRI blood oxygen level dependent (BOLD) signal change and neuronal activity.^4^ To assess the effective connectivity between significantly activated brain regions, a family of DCM are postulated and compared. We have used DCM to investigate the functional relationship between brain areas that showed activation in relation to IED that occurred during an EEG-fMRI scan.

These methods hold much promise in demonstrating the effective connectivity of cerebral areas involved in IED, in inferring the direction of propagation and the potential structural correlate of this connectivity that is not possible from EEG alone.

## METHODS

**Figure 1:**
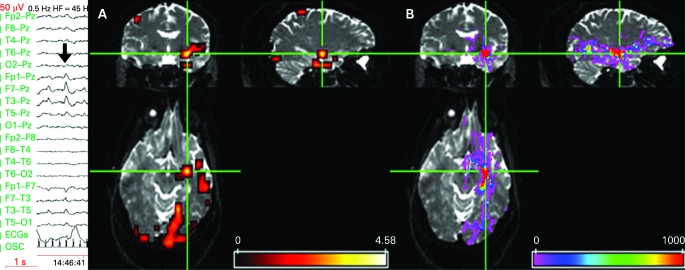
Left panel shows EEG recorded during fMRI scanning (10–20 system, upper 10 channels referential, lower 8 bipolar (OSC channel marks scanner gradient switching)) demonstrating left temporal activity in the delta/theta band with frequent intermixed spikes phase reversing at T3 (bold arrow). (A) Significant anterior temporal IED-related fMRI activations SPM{T} overlaid onto structural echo planar image (b = 0), showing left temporal and bilateral occipital fMRI activations. There were no significant deactivations. Scale represents z-score. (B) Tractography findings overlaid onto structural echo planar image, showing white matter connectivity to occipital and frontal areas. Cross hairs at left temporal fMRI maximum and the tractography seed region. The colour bar represents a measure of connection probability or connection confidence to the start point.

We studied a 23-year-old right-handed man with refractory complex partial seizures (onset age 7 years) lasting 1–5 minutes and occurring every other day. These comprised a brief aura of “abnormal feelings”, followed by an inability to vocalise, then unresponsiveness with right-arm posturing and subsequent bimanual automatisms. Secondarily generalised seizures occurred twice weekly, beginning with extension of the right arm, then asymmetrical clonic movements, right greater than left. Interictal EEG showed left anterior temporal excess delta activity and frequent left temporal spikes phase reversing at T3 (fig 1). Ictal video-EEG telemetry indicated seizure onset in the left temporal lobe, spreading rapidly to the right temporal lobe. MRI showed left hippocampal sclerosis. The patient declined surgical management.

The study was approved by the Joint Research Ethics committee of the National Hospital for Neurology and Neurosurgery, and written consent was obtained.

### MR imaging

A GE Horizon echospeed 1.5T MRI scanner (Milwaukee, USA) was used.

### EEG

Simultaneous EEG and fMRI were acquired over 35 minutes. Ten channels of EEG referenced to Pz in bitemporal chains and ECG were recorded using in-house recording equipment.^5^

### fMRI

Seven hundred BOLD sensitive echo planar images (EPI) (TE/TR 40/3000 (no gap), 21 × 5 mm, interleaved axial slices, field of view (FOV) 24 × 24 cm^2^, 64 × 64 matrix) were acquired over 35 minutes. SPM2 was used for all image preprocessing and analysis (http://www.fil.ion.ucl.ac.uk/spm).

### EEG-fMRI analysis

EEG spikes were visually identified and served as onsets for a general linear model (event-related fMRI analysis including convolution with a haemodynamic response function (HRF) and its temporal derivative). Realignment parameters were modelled as confounds. An F contrast was specified to test for significant spike-related BOLD signal changes; the computed SPM{F} was thresholded at p<0.05 level, corrected for multiple comparisons.

### Dynamic causal modelling

**Figure 2:**
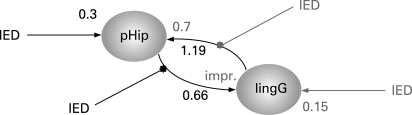
Two dynamic causal models were constructed, each comprising two structurally connected regions (6 mm diameter spheres), both showing interictal epileptiform discharge (IED)-correlated activation in the conventional SPM fMRI analysis: left parahippocampal gyrus (pHip) and left lingual gyrus (lingG). In both models, pHip and lingG were reciprocally connected. In model A (in bold print), IED acted on pHip and its connection to lingG (IED propagation from temporal to occipital lobe); in model B (grey print), IED acted on lingG and its connection to pHip (IED propagation from occipital to temporal lobe). DCM model comparison revealed strong evidence for model A over model B. In dynamic systems, coupling strength is expressed as a rate—typically, 0.5–1 Hz for regional activity. In both models, intrinsic connectivity was stronger from lingG to pHip (1.19 Hz in A, 0.7 in B) than vice versa (0.66 Hz in A and improbable in B). The induced response of IED on pHip was 0.3 Hz in model A and 0.15 Hz on lingG in model B. Combining both models in one to facilitate direct comparison (data not shown), the probability was 99.8% for IED, inducing a direct response in pHip, but only 61.8% in lingG.

We used DCM to assess effective connectivity.^6^ Neuronal activities were modelled using the known inputs (IED) and outputs (BOLD responses measured with fMRI). The specific models were that (i) changes in the states of the left lingual gyrus ([−20, −58, 1], table 1) depend on the activity of the left parahippocampal gyrus [−32, −1, −20], table 1) or (ii) vice versa. This dependency was parameterised by effective connectivity: (i) input parameters that describe how much brain regions respond to IED; (ii) intrinsic parameters that characterise effective connectivity among regions; and (iii) modulatory parameters that characterise changes in effective connectivity, here again due to IED. This third set of parameters, the modulatory effects, allows assessment of changes in coupling between the two brain regions. Two models were constructed (fig 2) based on the above GLM applied to alternate slices of the acquisition, resulting in an effective TR of 1.5 seconds, which is required for DCM. The likelihood of each model explaining the data was assessed using Bayesian statistics.^7^

**Table 1:** Results of EEG-fMRI analysis giving Talairach coordinates (obtained using software “mni2tal” (http://www.mrc-cbu.cam.ac.uk/Imaging/mnispace.html), Talairach labels (from Talairach Daemon (http://ric.uthscsa.edu/projects/talairachdaemon.html)) and Z scores of fMRI activations associated with interictal left temporal discharges, SPM{F}, corrected p<0.05; extent threshold 50

Region	Talairach coordinates	Cluster size (voxels)	Z score
Temporal lobe			
L parahippocampal gyrus	−32 −1 −20	1192	>8
L middle temporal gyrus	−59 −20 −14	299	6.40
L middle temporal gyrus	−51 8 −31	101	6.66
L middle temporal gyrus	−46 −62 3	231	6.11
L superior temporal gyrus	−65 −17 3	301	5.78
Parietal lobe			
L postcentral gyrus	−22 −31 72	1720	>8
R postcentral gyrus	48 −19 53	1204	7.77
Occipital lobe			
L lingual gyrus	−20 −58 1	6292	7.65
R inferior occipital gyrus	28 −91 −4	69	5.81

### Tractography

Whole-brain diffusion imaging data were acquired with a cardiac-gated single-shot spin-echo EPI sequence (TE = 95 ms, 96 × 96 matrix (128 × 128 reconstruction), 22 × 22 cm^2^ FoV, 60 contiguous 2.3 mm axial slices (whole brain coverage), maximum b value 1148 mm^2^ s^−1^ (δ = 34 ms, Δ = 40 ms, full gradient strength 22 mT/m), 54 non-colinear directions, 6 non-diffusion weighted (b = 0) volumes, acquisition time ∼25 min).^8^

We used the probabilistic index of connectivity (PICo) algorithm,^3^ ^9^ which was designed to incorporate multiple fibre populations to avoid ambiguities in voxels containing fibre crossings, to track the anatomical connections from the maximum temporal lobe activation obtained from the EEG-fMRI, thresholded at a connection probability of 0.01.^10^

The SPM{F} image from EEG-fMRI was resliced, reoriented and overlaid onto the b = 0 image. A region of interest (ROI) of 31 voxels (including both grey and white matter) in the left mesial temporal maximum BOLD activation was used as a seed for the PICo tractography analysis.

## RESULTS

There were 177 IEDs during the 35-minute EEG-fMRI session. Three distinct areas of activation were seen within the left temporal lobe, with maxima in the left parahippocampal gyrus, the left middle temporal gyrus and left superior temporal gyrus. Activations were also seen bilaterally in parietal and occipital lobes (L>R), including in the lingual gyrus (table 1 and fig 1A). Tractography demonstrated connectivity from the left temporal lobe to the left occipital and orbito-frontal frontal areas (fig 1B).

DCM showed stronger intrinsic effective connectivity from the lingual gyrus to the parahippocampal gyrus than the parahippocampal gyrus to the lingual gyrus. However, comparison of the two effective connectivity models showed much stronger evidence for the IED acting on the left parahippocampal and its connection to the lingual gyrus than for the reciprocal model (fig 2).

## DISCUSSION

Using EEG-fMRI, we found activations with left temporal spikes in the left temporal lobe, bilateral parietal and bilateral (L>R) occipital lobes. There were three areas of activation within the left temporal lobe, involving the parahippocampal/hippocampal formation, the superior temporal and middle temporal gyri. It has previously been demonstrated with EEG dipole modelling that separated sources can generate IEDs, with propagation from a mesial to a lateral temporal source.^11^

Areas of fMRI activation distant to the electro-clinical focus are often seen in EEG-fMRI studies.^1^ The clinical semiology, interictal EEG and ictal video EEG findings were consistent with left temporal onset, and there was no electro-clinical evidence to support occipital or parietal seizure onset. The DCM analysis indicated an intrinsic connectivity that was predominantly from lingual gyrus to parahippocampal gyrus. When temporal IEDs occurred, however, the connectivity from parahippocampal gyrus to lingual gyrus was strengthened, suggesting that this was the direction of propagation of the IED. This situation is different to that of an occipital focus propagating to the anterior medial temporal lobe.^12^

The concept of a network of epileptic activity rather than a single focal generator is well established, with much evidence being obtained from intracranial recordings.^13^ MRI methods, despite the indirect measure and much lower temporal resolution, have two key advantages: they are non-invasive; and they allow whole-brain imaging of the correlates of IEDs, as opposed to the limited spatial sampling of intracranial recordings.

Tractography has previously been used in healthy volunteers to demonstrate the anatomical connections from the anterior parahippocampal gyrus to the occipital lobe,^14^ reflecting the inferior longitudinal fasiculus (ILF).^15^ The ILF passes to the superior, middle and inferior temporal gyri on the lateral surface of the temporal lobe and medially to the uncus/parahippocampal gyrus close to the amygdala and hippocampus.^15^ MR-tractography is blind to the direction of neural propagation, and cannot distinguish between afferent and efferent pathways. The ILF probably represents the route of fast occipito-temporal transmission of visual inputs for associative processing (this direction of intrinsic connectivity was strongest in our DCM model), and temporo-occipital projections that modulate the processing of visual stimuli.

We suggest that the tractography analysis visualised the pathway of IED propagation from the parahippocampal gyrus to the occipital lobe. Structural connectivity between the three separate temporal activations was not seen on tractography—probably as a result of insufficient spatial resolution, and statistical constraints imposed by a single case.

Tractography seeded from the left mesial temporal fMRI maximum demonstrated strong structural connections not only to the left occipital lobe, but also to the left orbito-frontal cortex. The latter area did not activate with left temporal IED, reflecting the fact that IED propagation is along selected pathways and is not universal.^13^

The combination of tractography and fMRI with DCM offers a technique for investigating cerebral networks in health and disease, and for identifying the effective and functional connectivity between cerebral areas and the structural basis of this.
